# Fungal Laccases and Their Applications in Bioremediation

**DOI:** 10.1155/2014/163242

**Published:** 2014-05-15

**Authors:** Buddolla Viswanath, Bandi Rajesh, Avilala Janardhan, Arthala Praveen Kumar, Golla Narasimha

**Affiliations:** Applied Microbiology Laboratory, Department of Virology, Sri Venkateswara University, Tirupati 517 502, India

## Abstract

Laccases are blue multicopper oxidases, which catalyze the monoelectronic oxidation of a broad spectrum of substrates, for example, *ortho-* and *para-*diphenols, polyphenols, aminophenols, and aromatic or aliphatic amines, coupled with a full, four-electron reduction of O_2_ to H_2_O. Hence, they are capable of degrading lignin and are present abundantly in many white-rot fungi. Laccases decolorize and detoxify the industrial effluents and help in wastewater treatment. They act on both phenolic and nonphenolic lignin-related compounds as well as highly recalcitrant environmental pollutants, and they can be effectively used in paper and pulp industries, textile industries, xenobiotic degradation, and bioremediation and act as biosensors. Recently, laccase has been applied to nanobiotechnology, which is an increasing research field, and catalyzes electron transfer reactions without additional cofactors. Several techniques have been developed for the immobilization of biomolecule such as micropatterning, self-assembled monolayer, and layer-by-layer techniques, which immobilize laccase and preserve their enzymatic activity. In this review, we describe the fungal source of laccases and their application in environment protection.

## 1. Introduction

Fungi can exploit marginal living conditions in large part because they produce unusual enzymes capable of performing chemically difficult reactions [1, 2]. Interest in laccases has increased recently because of their potential use in the detoxification of pollutants and in bioremediation of phenolic compounds [3–5]. These fungal enzymes can convert wood, plastic, paint, and jet fuel among other materials into nutrients. Some of these enzymes have already been harnessed in pulp and paper processing and in the synthesis of fine chemicals [6]. Recent studies have suggested that lignin-degrading or white-rot fungi (decay caused by these species that gives wood a bleached appearance) such as* Phanerochaete chrysosporium* and* Trametes versicolor* could replace some of the chemical steps used in paper making [2, 7].

The use of enzymes for the treatment or the removal of environmental and industrial pollutants has attracted increasing attention because of their high efficiency, high selectivity, and environmentally benign reactions. Of these enzymes studied for such purposes extracellular fungal peroxidases, such as lignin peroxidase, manganese peroxidase, and fungal laccases are the two major classes of enzymes that have been evaluated for the removal of toxic phenolic compounds from industrial wastewater and the degradation of recalcitrant xenobiotics. Numerous reports have been published recently on the improvements of the production of these enzymes, such as discovery of new fungal strains, modification of growth conditions, use of inducers, and use of cheaper growth substrates such as agricultural and food wastes. The review of the literature given below is therefore an account relating to laccases and their production, purification, biochemical characterization, and their applications.

Laccase (EC 1.10.3.2,* p*-diphenol: dioxygen oxidoreductase) is one of a few enzymes that have been studied since the nineteenth century. Yoshida first described laccase in 1883 when he extracted it from the exudates of the Japanese lacquer tree,* Rhus vernicifera* [8]. In 1896 laccase was demonstrated to be a fungal enzyme for the first time by both Bertrand and Laborde [9]. Laccases of fungi attract considerable attention due to their possible involvement in the transformation of a wide variety of phenolic compounds including the polymeric lignin and humic substances [10]. Most lignolytic fungal species produce constitutively at least one laccase isoenzyme and laccases are also dominant among lignolytic enzymes in the soil environment. In addition, laccase-mediated delignification allows increasing the nutritional value of agroindustrial byproducts for animal feed or soil fertilizer [11].

The fact that they only require molecular oxygen for catalysis makes them suitable for biotechnological applications for the transformation or immobilization of xenobiotic compounds [12]. The major role of laccases in lignin and phenolic compound degradation has been evaluated in a large number of biotechnological applications such as dye degradation, bioremediation of some toxic chemical wastes (e.g., chlorinated aromatic compounds, polycyclic aromatic hydrocarbons, nitroaromatics, and pesticides) and biosensor developments [11–13]. Commercially, laccases have been used to delignify wood tissues, produce ethanol, and distinguish between morphine and codeine. Research in recent years has been intense, much of it elicited by the wide variety of laccases, their utility, and their very interesting properties. The current status of knowledge with regard to fungal laccase and their applications to protect environment is reviewed.

## 2. Distribution and Physiological Functions of Laccases

Laccases are common enzymes in nature and are found widely in plants and fungi as well as in some bacteria and insects [14]. The physiological functions of these biocatalysts, which can be secreted or intracellular, are different in the various organisms but they all catalyse polymerization or depolymerization processes [15]. As mentioned earlier, the first laccase was reported in 1883 from* Rhus vernicifera*, the Japanese lacquer tree from which the designation laccase was derived, and the enzyme was characterized as a metal containing oxidase [16]. This makes it one of the earliest enzymes ever described. Laccases have subsequently been discovered from other numerous plants [17] but the detection and purification of plant laccases are often difficult because crude plant extracts contain a large number of oxidative enzymes with broad substrate specificities [17], which is probably the reason why detailed information about the biochemical properties of plant laccase is limited. However,* Rhus vernicifera* laccase is an exception and has been extensively studied, especially with regard to its spectroscopic properties [18].* R. vernicifera* laccase has also widely been used in investigations of the general reaction mechanism of laccases [19, 20]. Plant laccases are found in the xylem, where they presumably oxidize monolignols in the early stages of lignification [21], and also participate in the radical-based mechanisms of lignin polymer formation [22]. In addition, laccases have been shown to be involved in the first steps of healing in wounded leaves [23]. However, the occurrence of laccases in higher plants appears to be far more limited than in fungi [24, 25].

Only a few bacterial laccases have been described hitherto. The first bacterial laccase was detected in the plant root-associated bacterium “*Azospirillum lipoferum*” [26], where it was shown to be involved in melanin formation [27]. An atypical laccase containing six putative copper-binding sites was discovered from* Marinomonas mediterranea,* but no functional role has been assigned to this enzyme [28].* Bacillus subtilis* produces a thermostable CotA laccase which participates in pigment production in the endospore coat [29]. Laccases have also been found from* Streptomyces cyaneus* [30] and* Streptomyces lavendulae* [31]. Although there are also some other reports about laccase activity in bacteria, it does not seem probable that laccases are common enzymes from certain prokaryotic groups [32]. Bacterial laccase-like proteins are intracellular or periplasmic protein [10]. Laccases producing bacteria from different environmental sources with their possible physiological functions of laccase are given below.* B. licheniformis* is a novel melanogenic soil bacterium isolated from soil, which protects strain from UV light and the oxidants [33]. It is involved in dimerization of phenolic acids [34].* Bacillus* endospores producing laccase were isolated from soil and the enzyme involved in phenol degradation [5, 35].

Laccase activity has been demonstrated in many fungal species belonging to ascomycetes and basidiomycetes, and the enzyme has already been purified from many species. There are many records of laccase production by ascomycetes. Phytopathogenic ascomycetes like* Melanocarpus albomyces *[36],* Cerrena unicolor *[37],* Magnaporthe grisea *[38],* Trametes versicolor *[14],* Trichoderma reesei *[39], and* Xylaria polymorpha *[40]are examples for laccase production and the enzyme was purified. Besides, in plant pathogenic species, laccase production was also reported for some soil ascomycete species from the genera* Aspergillus, Curvularia,* and* Penicillium *[41] as well as some fresh water ascomycetes [42]. Yeasts are a physiologically specific group of both ascomycetes and basidiomycetes. Until now, laccase was only purified from the human yeast pathogen* Cryptococcus* (*Filobasidiella*)* neoformans*. This yeast produces true laccase capable of oxidation of phenols and aminophenols and unable to oxidize tyrosine [43]. The enzyme is tightly bound to the cell wall and contributes to the resistance to fungicides [44]. Among physiological groups of fungi, laccases are typical of the wood-rotting basidiomycetes, which cause white rot, and a related group of litter-decomposing saprotrophic fungi, that is, the species causing lignin degradation. Almost all species of white-rot fungi were reported to produce laccase in varying degrees and the enzyme has been purified from many species [45].

The majority of laccases characterized so far have been derived from white-rot fungi which are efficient lignin degraders [46]. Many fungi contain several laccase-encoding genes, but their biological roles are mostly not well understood [47].* Agaricus bisporus* [48],* Botrytis cinerea *[49],* Coprinus cinereus *[50],* Phlebia radiata *[51],* Pleurotus ostreatus *[52], and* Trametes versicolor *[53] were some examples of basidiomycetes that produce laccases. In addition to plants, bacteria, and fungi, laccases or laccase-like activities have been found in some insects, where they have been suggested to be active in cuticle sclerotization [54]. Recently, laccases have been represented as candidates for lignin modification enzymes (“lignases”) in termites. Predominant laccase activities against DMP and ABTS were detected in the fungus combs and fungi isolated from the nests of three genera of fungus-growing termites, that is,* Macrotermes*,* Odontotermes*, and* Microtermes* [55–57].

## 3. Screening of Fungal Species

Screening of laccase producing fungal species and their variants is important for selecting suitable laccase producing organisms. For this reason one usually relies on the use of inexpensive, rapid, and sensitive testing methods. The screening strategy must aim to identify fungal strains and enzymes that will work under industrial conditions [58]. Discovery of novel laccases with different substrate specificities and improved stabilities is important for industrial applications. Fungi that produce laccase have been screened for either on solid media containing coloured indicator compounds that facilitate the visual detection of laccase production [59] or with liquid cultivations monitored with enzyme activity measurements [60]. The use of coloured indicators is generally simpler as no sample handling and measurement are required. As laccases oxidize various types of substrates, several different compounds have been used as indicators for laccase production.

The traditional screening reagents tannic acid and gallic acid [61] have nowadays mostly been replaced with synthetic phenolic reagents, such as guaiacol and syringaldazine [59, 62], or with the polymeric dyes Remazol Brilliant Blue R (RBRR) and Poly R-478 [63]. RBRR and Poly R-478 are decolourized by lignin degrading fungi [64] and the production of laccase is observed as a colourless halo around microbial growth. With guaiacol a positive reaction is indicated by the formation of a reddish brown halo [59], while with the tannic acid and gallic acid the positive reaction is a dark-brown coloured zone [61]. Kiiskinen et al. [46] screened novel laccase producing fungi by a plate method based on polymeric dye compounds, guaiacol and tannic acid.

## 4. Cultural and Nutritional Conditions for Laccase Production

Culture conditions and medium composition play a major role in enzyme expression. Laccase production by fungi is strongly affected by many fermentation parameters such as time of cultivation, stationary or submerged cultures, organic or inorganic compound concentrations, inducer concentration [65], aeration [66], and degradation or activation by protease [67]. Laccases are generally produced during the secondary metabolism of white-rot fungi growing on natural substrate or in submerged culture [2]. Physiological demands vary among white-rot fungi and considerable research has been done on the influence of agitation, pH, temperature, carbon, nitrogen sources, and microelements and their levels.

Dong et al. [68] compared laccase production by* Trametes gallica* on twelve media under static or shaking conditions. They concluded that twelve culture media, in addition to static and shaking conditions, show great influence on amount and pattern of laccase isoenzymes from* Trametes gallica*. Gayazov and Rodakiewicz-Nowak [69] reported faster laccase production under semicontinuous cultivation with high aeration and culture mixing compared to static conditions. When using conical flasks for cultivation it should be baffled to ensure a high oxygen transfer [70]. Similarly, Piscitelli et al. [71] described the influence of various physiological factors on laccase formation in a number of white-rot fungi. Refined media are necessary for obtaining large amount of laccases that may be used in biochemical analysis and industrial application. Extracellular laccases of white-rot fungi exist in isozymes that may be inducible or constitutive.* Ganoderma lucidum* produced more than three laccase isozymes in liquid culture [72].* Pleurotus pulmonarius* produced three laccase isozymes among which the* lcc*1 and* lcc*2 isoforms were produced in noninduced cultures, while* lcc*3 was found only in induced-culture filtrates [73]. At least seven laccase isozymes were found in the basidiomycete CECT 20197 [74]. Three constitutive and four induced laccase isozymes were found in* Marasmius querocophilus *strain 17 [75]. At least nine constitutive laccase isozymes were described for* Pleurotus *sp. [76]. The pattern of isozymes has been successfully applied in the identification of a number of different microorganisms, particularly fungi such as ectomycorrhiza [77], deuteromycetes [78], and basidiomycetes [79, 80].

A common technique for comparison of isozyme patterns from different sources is the use of zymograms [81]. Praveen et al. [82] found that the production of high titres of the laccase enzyme was not dependent on high biomass yields. But laccase production was found to be highly related to the conditions of cultivation of the fungus [82, 83] and media supporting high biomass did not necessarily support high laccase yields [84]. The synthesis and activity of the laccase were controlled during growth and can play an important role in pigment and fruiting body formation [8, 85]. Buswell et al. [86] reported that the production of laccase was strongly affected by the nature and amounts of nutrients, especially nitrogen and trace elements in the growth medium. Laccases were generally produced in low concentrations by laccase producing fungi [87], but higher concentrations were obtainable with the addition of various supplements to media [71]. Laccase production by* Phanerochaete chrysosporium* was not detected in low or high nitrogen medium with glucose as the carbon source but was produced when the organism was grown on low or high nitrogen medium with cellulose as the carbon source [88], whereas, in the white-rot fungus* Ganoderma lucidum,* higher levels of laccases were produced in high nitrogen medium with glucose as the carbon source [89]. Ligninolytic systems of white-rot fungi were mainly activated during the secondary metabolic phase and were often triggered by nitrogen concentration [86] or when carbon or sulfur became limited [83]. The addition of xenobiotic compounds such as xylidine, lignin, and veratryl alcohol was known to increase and induce laccase activity [84]. Towards maximization of laccase secretion with culture additives, Sinegani et al. [90] studied laccase production by* Aspergillus terreus, Armillaria* sp.,* Polyporus* sp., and* Phanerochaete chrysosporium* in liquid culture media treated with N-ethyl aniline, N,N-dimethyl aniline and* para*-bromoaniline as a laccase inducer.

Optimization of the production medium plays a major role in higher laccase production. The Taguchi approach of OA DOE constitutes a simple methodology that selects the best conditions producing consistent performance. This approach led to an increase in laccase yield to 820 U/L from 485 U/L. The increased production of laccase was also confirmed by the dye decolorization experiment, which showed an increased decolorization of reactive blue 221 from 45% to 84.6% in the same unit volume [91].

### 4.1. Influence of pH on Laccase Production

The pH of the culture medium is critical and plays a significant role in the growth and laccase production of the organism. There is not much information available on the influence of pH on laccase production, but when fungi are grown in a medium with pH 5.0 laccase will be produced in excess [8]. Most reports indicated initial pH levels set between pH 4 and 6 prior to inoculation, but the levels were not controlled during most cultivations [87, 92]. The optimum pH of laccase production, as reported in many fungi, falls between 5.0 and 6.0 [14, 93, 94]. Maximum titres of laccase and biomass were observed in the medium adjusted to pH 6.0 by* Fomes sclerodermeus*, white-rot basidiomycetes. The optimal range for the laccase isoforms secreted by* Trametes pubescens *fungal strain has been reported between pH 3.0 and 4.5, potentially indicating that laccase may be produced and function optimally under conditions that are not favourable to growth [95]. Laccases from fungi have been found in wide applications ranging from the pharmaceutical sector to the pulp and paper industry, but eukaryotic laccases generally prefer low pH for better functioning. In contrast, bacterial laccases can act and are more stable at wider pH range [96, 97]. With the advantage of immense environmental adaptability and biochemical versatility, prokaryotes deserve to be studied for their possible applications in industry as well as in medical science [5].

### 4.2. Influence of Temperature on Laccase Production

Temperature, like any other physical parameters, plays a vital role in growth and laccase production of the organism. It has been found that the optimal temperature for fruiting body formation and laccase production is 25°C in the presence of light but 30°C for laccase production when the cultures are incubated in the dark [8]. In general the fungi were cultivated at temperatures between 25°C and 30°C for optimal laccase production [14]. When cultivated at temperatures higher than 30°C, the activity of laccase was reduced [98]. The wood-decaying basidiomycete* Steccherinum ochraceum* isolate 1833 was reported to produce three highly thermostable laccase isoforms with maximum activities in the region 75–80°C [99, 100]. Therefore, it is proved that optimum production of laccase can differ greatly from one strain to another.

### 4.3. Influence of Carbon on Laccase Production

Carbon is a part of all living organisms. Breakdown of carbon sources liberates energy, which is utilized by the organism for growth and development. The most readily usable carbon source by white-rot fungi is glucose [101]. Collins and Dobson [102] reported that glucose at 10 g/L enhanced the growth and laccase production by* Coriolus versicolor*. In* Trametes versicolor*, glucose at higher concentration (20 g/L) favoured laccase production [14]. In* Ganoderma lucidum*, glucose at 20 g/L increased the mycelial growth but at 10 g/L favoured expression of enzyme [103]. The maximum titres of extracellular laccase in cultures of* Lentinula edodes *and* Grifola frondosa *were grown in liquid medium with 10 g/L glucose [104, 105]. Glucose at 5 g/L in the liquid medium supported laccase production by* Trametes gallica* [68]. Maximum laccase production was obtained using response surface methodology with glucose (15.21 g/L) as the carbon source for* Pleurotus florida* NCIM 1243 [106].

Among several carbon sources tested, malt extract turned out to be the best carbon source in the medium for pronounced laccase production by* Phlebia floridensis*,* P. brevispora, P. radiata, *and* P. fascicularia *[107]. D'Souza-Ticlo et al. [108] screened different carbon sources for maximum laccase production by* Botryosphaeria* sp. They have screened glucose, fructose, galactose, galacturonic acid, xylose, lactose, sucrose, mannitol, pectin, and inulin and found increased laccase production with most carbon sources studied except inulin and galacturonic acid. Revankar and Lele [109] obtained highest laccase activities with* Trametes versicolor *MTCC 138 using different carbon sources, namely, glucose, fructose, sucrose, lactose, starch, and glycerol. They observed a 3-fold increase of laccase production when glucose was used instead of fructose, and starch further improved laccase production by 12%. The carbon source mannitol increased laccase enzymatic activity to 115.62% in* Ganoderma lucidum strain* 7071-9 in* Pichia pastoris* at a concentration of 1 mM but had no effect at 0.1 mM [110].

### 4.4. Influence of Nitrogen Sources on Laccase Production

Laccases of white-rot fungi are mainly activated during the secondary metabolic phase of the fungus and are often triggered by nitrogen depletion [111], but it was also found that in some strains nitrogen concentrations had no effect on laccase activity [112]. These contradictory observations were ascribed to differences between the strains of* Phanerochaete chrysosporium* and* Lentinus edodes *[86]. Buswell et al. [86] found that laccases were produced at high nitrogen concentrations although it is generally accepted that a high carbon to nitrogen ratio is required for laccase production. Laccase was also produced earlier when the fungus was cultivated in a substrate with a high nitrogen concentration and these changes did not reflect differences in biomass. Heinzkill et al. [83] also reported a higher yield of laccase using nitrogen rich media rather than the nitrogen limited media usually employed for induction of oxidoreductase. In another study by [113], a rise in nitrogen concentration (from 0.25 to 2.0 g/L) enhanced laccase synthesis yield. Higher nitrogen levels are often required in order to enhance laccase production [114] but with certain fungi nitrogen-limited culture conditions simulate the formation of laccase enzyme [111, 115]. They stated that the culture parameter with the most deleterious effect on extracellular enzyme activity was of high level nitrogen concentration.

The optimum nitrogen concentration for obtaining the highest laccase activity from* Pycnoporus sanguineus* (820 mU mL^−1^) is provided by a sucrose-asparagine medium containing 5 times as much asparagine as Kirk's medium; in fact, such a medium provided a 2.5 times higher laccase activity than reference medium. These conditions yielded maximum laccase activity [116]. Laccase was best produced at the concentration of 100 mg NH_4_Cl and 50 mg malt extract with gross yield of 0.725 U/mL. [117]. The nitrogen source that improved laccase synthesis to the greatest extent was peptone (1.8-fold increase) [95]. Revankar and Lele [109] obtained highest laccase activities by* Trametes versicolor* MTCC 138 using a complex nitrogen source (yeast extract). Leatham and Kent Kirk [111] screened different nitrogen sources, namely, KNO_3_, glutamic acid, glycine, beef extract, and corn steep liquor, and found that glutamic acid with low concentration yielded higher amounts of laccase.

### 4.5. Influence of Aromatic Compounds on Laccase Production

Low molecular weight aromatic compounds have shown significant influence on the growth and activity of lignocellulolytic microorganisms [118]. Several compounds with a methylated* p*-phenolic group are the products of ferulic and syringic acid metabolism in* Phanerochaete chrysosporium *[119]. Aromatic compounds which are structurally related to lignin, such as xylidine, ferulic acid, and veratric acid, are routinely added to fungal cultures to increase laccase production [105, 120]. Xylidine is known to increase laccase transcription in* Trametes villosa *[121],* Trametes versicolor *[122], and* P. sajor-caju *[123]. It has been reported that one of the possible functions for fungal laccase is the polymerization of toxic aromatic compounds formed during the degradation of lignin [8]. A dark precipitate was observed in xylidine-induced cultures of* T. versicolor* and has been suggested that it may represent a laccase polymerized form of aromatic compounds [122]. Earlier studies on white-rot fungi have shown that methylation of lignin-related aromatics inhibits fungal growth only at higher concentrations (5 and 10 mM) with stimulation occurring at 1 mM concentration [124]. Veratryl (3, 4-dimethoxybenzyl) alcohol is an aromatic compound known to play an important role in the synthesis and degradation of lignin.

The addition of veratryl alcohol to cultivation media of many white-rot fungi has resulted in an increase in laccase production [125]. Some of these compounds affect the metabolism or growth rate [126] while others, such as ethanol, indirectly trigger laccase production [127]. Many of these compounds resemble lignin molecules or other phenolic chemicals [105]. There are many reports describing the different effects of aromatic compounds on laccase activity. Highest laccase activity was observed in* Botryosphaeria rhodina* when veratryl alcohol was added to the nutrient medium at the beginning of fermentation [128]*. R. lignosus* shows maximum laccase activity with compound with phenylhydrazine [128]. In a study by Elisashvili and Kachlishvili, 2, 4, 6-trinitrotoluene (TNT) supplemented medium at appropriate concentration significantly accelerated* Cerrena unicolor *laccase production and 4-fold increased laccase specific activity [129]. Xylidine is known to increase laccase transcription in* Trametes villosa* [120] and in* Trametes versicolor* [121, 130]. The addition of veratryl alcohol to cultivation media of many white-rot fungi has resulted in an increase in laccase production [124, 130]. The aromatic compound hydroquinone increased 3-fold* T. versicolor* laccase activity while decreasing 2- and 8-fold yields of MnP and endoglucanase [131]. Hadibarata et al. [132] have proved the importance of extracellular laccase of* Armillaria *sp. F022 in the transformation of anthracene into anthraquinone, benzoic acid, and other products such as 2-hydroxy-3-naphthoic acid and coumarin.

### 4.6. Influence of Amino Acids on Laccase Production

Laccase production by white-rot fungi is strongly affected by the presence of amino acids in the media [68]. Various amino acids and their analogues have shown stimulatory as well as inhibitory effects on laccase production by* Cyathus bulleri* [133]. According to this study, DL-methionine, DL-tryptophan, glycine, and DL-valine stimulated laccase production, while L-cysteine monohydrochloride completely inhibited the enzyme production. Dong et al. [68] used amino acid mixture that contained the following amino acids: L-arginine, L-histidine, L-valine, L-threonine, L-isoleucine, L-tyrosine, L-methionine, L-serine, L-asparagine, L-lysine, L-aspartic acid, L-tryptophan, and L-cysteine, at 1% (w/v) concentration. This amino acid mixture favoured the laccase production by* Trametes gallica*. It is possible that amino acids act as inducer for laccase production by many white-rot fungi [68, 133]. Sun et al. [110] reported that all the six amino acids (alanine, histidine, glycine, arginine, aspartate, and phenylalanine) at 1 mM concentration increase the catalytic ability of the laccase enzyme from* Ganoderma lucidum strain *7071-9 when expressed in* Pichia pastoris*. Amino acid tryptophan also induces laccase production in* Crinipellis* sp. RCK-1 [134].

### 4.7. Influence of Copper on Laccase Production

Copper is an indispensable micronutrient for most living organisms and copper requirements by microorganisms are usually satisfied by low concentrations of the metal [105]. In its free form cupric ion at higher concentration is extremely toxic to microbial cells. The binding and uptake of copper in fungi usually comprise two phases: metabolism-independent surface binding followed by an energy-dependent metal influx [135]. The stimulatory effect of copper on laccase synthesis was also effective for several other basidiomycetes and hence could be used as a simple method to improve the production of this enzyme [136]. Using northern blot analysis it was determined that increased production of laccase activity would be obtained in the copper supplemented cultures of* Pleurotus ostreatus* [65].

Copper has been reported to be a strong laccase inducer in several species, for example,* Neurospora crassa* [137],* Trametes versicolor *[122],* Phanerochaete chrysosporium* [138],* Panus osteratus* [65],* Pleurotus sajor-caju* [123],* Trametes trogii* [101],* Volvariella volvacea* [139],* Lentinula edodes* [104], and* Grifola frondosa* [105]. Huber and Lerch [136] reported that* Trametes pubescens* grown at 2.0 mM CuSO_4_ exhibited high laccase activity (65 U/mL) and using western blot analysis they further demonstrated that the synthesis of the laccase protein was linked to the presence of copper ions in the culture medium. In addition, both the time and the concentration of copper supplementation were important for obtaining high levels of laccase. According to a recent study on the white-rot fungus* Trametes trogii* [101], the addition of copper strongly stimulated lignolytic enzyme production, and higher decolorization of polymeric dyes-poly R-478 was observed as well. However, higher copper concentrations (500 mM) inhibited the growth and notably decreased manganese peroxidase production although they did not affect secretion of laccase [101]. The addition of low concentrations of copper to the cultivation media of laccase producing fungi stimulated laccase production [140]. Palmieri et al. [65] found that the addition of 150 *μ*M copper sulphate to the cultivation media can result in a fiftyfold increase in laccase activity compared to a basal medium. Copper has been reported to be a strong laccase inducer in several species, for example,* Neurospora crassa* [136],* Paecilomyces* sp. WSH-L07 [141],* Shiraia bambusicola* strain GZ11K2 [142],* Trametes trogii TEM H*2 [143],* Pleurotus* florida* NCIM* 1243 [106],* Peniophora* sp. [144], and so forth.

## 5. Laccases Inducers

The promoter regions of the genes encoding for laccase contain various recognition sites that are specific for xenobiotics and heavy metals [145]. These xenobiotics and heavy metals can bind to the recognition sites of the gene when present in the medium and induce laccase production. White-rot fungi were very diverse in their responses to tested inducers for laccase. The addition of certain inducers can increase the concentration of a specific laccase or induce the production of new isoforms of the enzyme [146]. Some inducers interact variably with different fungal strains. Lu et al. [147] found that the addition of xylidine as inducer had the most pronounced effect on laccase production. The addition of 10 *μ*M xylidine after 24 h of cultivation gave the highest induction of laccase activity and increased laccase activity by ninefold. At higher concentrations the xylidine had a reduced effect probably due to toxicity. Laccase offers protection for the fungus against toxic phenolic monomers of polyphenols [147]. Dhawan and Kuhad [127] investigated the inducing effect of alcohols on the laccase production by* Trametes versicolor. *The enhanced laccase activity was comparable to those obtained using 2, 5-xylidine and veratryl alcohol [74]. It was hypothesized that the addition of ethanol to the cultivation medium caused a reduction in melanin formation. The monomers, when not polymerised to melanin, then acted as inducers for laccase production [127]. The addition of ethanol as an indirect inducer of laccase activity offers a very economical way to enhance laccase production. Garzillo et al. [148] found that there was a strong correlation between hyphal branching and the expression and secretion of laccase. The addition of cellobiose can induce profuse branching in certain* Pycnoporus* species and consequently increase laccase activity [148]. The addition of cellobiose and lignin can increase the activity of extracellular laccases without an increase in total protein concentration [149].

An important distinction may be drawn between laccases; they may either be inducible or constitutively expressed [150]. The constitutive, or noninducible, group does not react readily to dissolved compounds that exhibit properties similar to their substrates, and no inducer producing significant improvements in their yield has as yet been isolated. Single inducers may not elicit the desired response in laccase production, and a complex mixture of inducers may be required [151].

Several compounds may elicit a positive response on laccase production; these compounds known as inducers which include the metal ions, copper or cadmium [114], cycloheximide [152], and low molecular weight aromatic or organic acids, such as veratric acid [122] and ferulic acid [153] as well as other phenolic or aromatic compounds such as 2, 5-xylidine [154] and veratryl alcohol [155]. Soden and Dobson [122] proved that Cu^2+^ ions have the ability to induce laccase production by forming an integral prosthetic group. In the same way, natural substrates such as aromatic/phenolic compounds and lignin derivatives such as veratryl alcohol and 2, 5-xylidine induced laccase production [53]. There is evidence in* Trametes versicolor *that these compounds cause an increase in mRNA levels, but only copper was involved in increasing laccase mRNA translation [122]. The exact action of inducers is, however, unknown. It has been demonstrated that fungi may possess several isozymes of laccase encoded by several laccase genes, and these may be differentially regulated [122]. It has further been suggested that the action of certain inducers may be a direct result of their toxicity to the fungus and the capability of laccase to polymerize and detoxify them [156]. The use of inducers does, however, suffer from several disadvantages including their toxicity and the extra expense associated with the addition of an inducer. The white-rot fungus* Trametes* sp. AH28-2 can synthesize extracellular laccase by induction in cellobiose-based liquid culture medium [157]. Both yields and composition of laccase isoenzymes, produced by* Trametes* sp. AH28-2, would be quite different with induction by different small-molecule aromatic compounds,* o*-toluidine, guaiacol, and 3, 5-dihydroxytoluene, which affected microbial growth and the synthesis of laccase isoenzymes differentially [157].

It has been suggested that the addition of veratryl alcohol may not elicit an inductive effect; rather it may act as a protective agent against inactivation by hydrogen peroxide produced endogenously by the fungus [158], thereby indirectly eliciting a higher enzyme production. The addition of surfactants or detergents, for example, Tween 20 or 80, has resulted in higher yields of ligninolytic enzymes in certain fungi. There is evidence that these detergents result in higher permeability of oxygen and extracellular enzyme transport through the cell membranes of fungi [159]. Effective induction of laccase from* Pleurotus florida* with anionic and cationic surfactants has been demonstrated [160].

In spite of an initial inhibitory effect on mycelial growth, ethanol was shown to be a very strong inducer for laccase expression by* Pycnoporus cinnabarinus* [161]. Shankar and Shikha [144] reported that veratryl alcohol induced maximum laccase production giving 6.07 U/mL laccase activity by* Peniophora* sp., whereas 0.5 mM xylidine was used as an inducer to optimized production of laccase by* Coriolopsis caperata* RCK2011 under solid state fermentation [162]. Xylidine is the most widely reported inducer of laccase production and enhanced laccase specific production by 4-fold in* Coriolopsis polyzona* [163].

There have been many studies regarding the effects of inducers using a plethora of fungal genera, species, and even strains. Differences in laccase stimulation were already observed in very early studies more than half a century ago. Ethanol has improved laccase synthesis significantly when used as a carbon source [160] for a monokaryotic strain* Pycnoporus cinnabarinus*. Later work by this group indicated that ethanol improved gene expression and inhibited protease activity, thereby playing an important regulatory role in laccase production by the fungus [164].

## 6. Purification and Biochemical Properties of Laccases

Production of extracellular laccase is a common feature of many fungi, particularly those associated with wood decay or the terminal stages of decomposition of leaf litter. Current knowledge about the structure and biochemical properties of fungal laccase proteins is based on the study of purified proteins. More than 100 laccases from various fungi have been purified and characterized by researchers for more than 35 years [10]. Most of the white-rot fungi produce laccase in multiple isoforms [65]. Several purification steps are required to obtain a preparation free of both pigment and other contaminant proteins. Multiple steps like ultrafiltration, precipitation using ammonium sulphate or organic solvents, and ion exchange and size exclusion chromatography have been used for the purification of laccases from the culture filtrate. Typical fungal laccase is a protein of approximately 60–70 kDa with acidic isoelectric point around 4.0 [10].

Several laccase isoenzymes have been detected in many fungal species. More than one isoenzyme is produced in most white-rot fungi [165]. This has been demonstrated by* p-*phenylenediamine staining the laccase activity in all tested wood rot fungi after isoelectric focusing. All tested species, namely,* Coprinus plicatilis, Fomes fomentarius, Heterobasidion annosum, Hypholoma fasciculare, Kuehneromyces mutabilis, Leptoporus litschaueri, Panus stipticus, Phellinus igniarius, Pleurotus corticatus, P. ostreatus, Polyporus brumalis, Stereum hirsutum, Trametes gibbosa, T. hirsuta, *and* T. versicolor*, exhibited the production of more than one isoenzyme, typically with pI in the range of pH 3 to 5 [10]. The white-rot fungus* P. ostreatus* produces at least eight different laccase isoenzymes, six of which have been isolated and characterized [166, 167]. The production of laccase isoenzymes in* P. ostreatus* is regulated by the presence of copper, and the two dimeric isoenzymes have only been detected in the presence of copper [167]. Isoenzymes of laccase with different molecular weight and pI were also detected in the litter-decomposing fungus* Marasmius quercophilus* [168]. A study with 17 different isolates of this fungus showed that the isoenzyme pattern was consistent within different isolates. Moreover, all isolates showed the same isoenzyme pattern (one of the three laccase bands on SDS PAGE) after induction of laccase with different aromatic compounds [169].

The catalytic action of an enzyme is quantitatively described by the Michaelis constant *K*_*M*_ and the catalytic efficiency constant *k*_cat_. These constants have been measured for a large number of laccases, and rather great variance can be observed among them (Table 1). The *K*_*M*_ values of laccases are generally in the range of 2.5 *μ*M depending on the enzyme source and the reducing substrate (Table 1). The lowest *K*_*M*_ values have been measured with syringaldazine, which is a dimer of two molecules of 2, 6-dimethoxyphenol linked by an azide bridge. Either the azide bridge or the dimer form is apparently beneficial for the affinity of syringaldazine to laccases because the *K*_*M*_ values measured for monomeric 2, 6-dimethoxyphenol are generally higher than those obtained with syringaldazine (Table 1). The comparison of *K*_*M*_ values also shows that laccases from different source organisms have different substrate preferences [170]. The specificity for oxygen is less dependent on the enzyme, and *K*_*M*_ values of 20–50 *μ*M for O_2_ have been reported for several laccases [171, 172].

Very significant variance has also been observed in the catalytic efficiencies (*k*_cat_) of various laccases. Differences as high as 3500-fold can be seen in the *k*_cat_ values between different laccases with the same substrates (Table 1). On the other hand, the *k*_cat_ values for a single laccase do not generally differ more than 2–10-fold between different substrates, which reflects the fact that *k*_cat_ describes the rate of the electron-transfer reactions taking place inside the enzyme after substrate binding [172]. However, the variance in assay conditions must always be taken into account when the catalytic constants measured in different laboratories are compared. The constants in Table 1 have been measured under varying pH, ionic strength, and temperature conditions and by using different protein concentrations, all of which have a great effect on the results. In addition, different molar extinction coefficients for oxidation products have sometimes been used in spectrophotometric assays because the nature of the actual oxidation products is often complex or poorly understood. This affects particularly the numerical values of *k*_cat_.

In addition to the kinetic constants, the catalytic performance of laccases by catalytic activity and stability in different pH and temperature conditions has been described. The pH activity profiles of laccases are often bell-shaped, with optima around 4.6, when measured with phenolic substrates [50]. The decrease in laccase activity in neutral or alkaline pH values is affected by increasing hydroxide anion inhibition because, as a small anion, hydroxide ion is also a laccase inhibitor. On the other hand, increasing pH decreases the redox potential of the phenolic substrate, making the substrate more susceptible to oxidation by laccase [173]. Oxidation of nonphenolic substrates, such as ABTS, does not involve proton exchange, and therefore nearly monotonic pH activity profiles with highest activities at pH values of 2.3 are obtained [174]. In contrast to their activity, the stability of laccases is generally the highest at pH values around 8-9 [170, 175]. Temperature stabilities of laccases vary considerably, depending on the source organism. In general, laccases are stable at 30–50°C and rapidly lose activity at temperatures above 60°C [176, 177].

## 7. Molecular Biology of Laccases

The first laccase genes were isolated and sequenced about 18 years ago from the fungi* Neurospora crassa *[178],* Aspergillus nidulans *[179, 180], and* Phlebia radiata *[181]. Since then, sequencing of laccase genes has increased considerably. However, the number of laccase genes of which the corresponding protein products have been experimentally characterized is significantly lower. To date, there are about 20 such enzymes, most of which are fungal laccases (Table 2). A typical laccase gene codes for a protein of 500–600 amino acids and the molecular weights of laccases are usually in the range of 60 to 90 kDa when determined by SDS-PAGE (Table 2). Difference between the molecular weight predicted from the peptide sequence and the experimentally obtained molecular weight is caused by glycosylation, which typically accounts for about 10–20% of the total MW [182]. The isoelectric points of microbial laccases are generally around 3.6 (Table 2). Several fungal genomes contain more than one laccase gene [121]. The expression levels of different laccase genes typically depend on cultivation conditions [123]. For example, high nitrogen content of the medium has been shown to induce transcription of laccase genes in the Basidiomycete I-62 (CECT 20197) and in* Pleurotus sajor-caju *[123].

Copper is also often a strong inducer of laccase gene transcription, and this has been suggested to be related to a defense mechanism against oxidative stress caused by free copper ions [182]. In addition to copper, other metal ions such as Mg^2+^, Cd^2+^, or Hg^2+^ can also stimulate laccase expression [182]. Certain aromatic compounds that are structurally related to lignin precursors, such as 2, 5-xylidine or ferulic acid, have also been shown to increase laccase gene transcription in* Trametes villosa*,* Trametes versicolor, *and* Pleurotus sajor-caju* [123]. On the other hand,* Trametes villosa* and* Pleurotus sajor-caju* have also been shown to contain constitutively expressed laccase genes, and this may be related to different physiological roles of the various laccases in the fungi [123]. A clear understanding of expression laccase gene may lead to overproduction of laccase enzyme.

### 7.1. Heterologous Production of Laccases

The natural hosts produce very low yields of laccases for commercial purposes. Therefore, to improve the production, the cloning of laccase gens and heterologous expression are employed. Recent advances in the field of genetic engineering have allowed the development of efficient expression vectors for the production of functional laccase. Laccase gene was cloned in the most commonly used organisms,* Pichia pastoris* [183],* Aspergillus oryzae* [184],* A. niger *[185, 186],* A. nidulans *[187],* Trichoderma reesei *[188], and* Yarrowia lipolitica *[189]. Laccase production levels have often been improved significantly by expression in heterologous hosts, but the reported levels have still been rather low for industrial applications (Table 3). The common problems associated with heterologous expression of fungal enzymes are incorrect folding and inefficient codon usage of expression organisms, resulting in nonfunctional or low yields of enzyme. The incorrect substitution of carbohydrate residues during glycosylation of proteins, which is due to preferential utilization of specific carbohydrates by the expression organism, may pose an additional problem to heterologous expression. These problems are being overcome by using more advanced organisms as expression vectors whose codon usage and molecular folding apparatus are suitable for correct expression of these proteins.

Production of heterologous laccase has often been improved by varying the cultivation conditions. For example, better production of heterologous laccase has been achieved in yeast systems by controlling the pH of the culture medium and by lowering cultivation temperatures [190, 191]. Buffering of the culture medium to maintain the pH above 4 has been proposed to be important in stability of secreted laccases and inactivation of acidic proteases [190], whereas lowered cultivation temperatures may result in better production due to improved folding of heterologous proteins [192]. In addition, over expression of Sso2p, a membrane protein involved in the protein secretion machinery [192]. Larsson et al. has been shown to improve heterologous laccase production in* S. cerevisiae* [193, 194].

The addition of copper to the culture medium has also proved to be important for heterologous laccase production in* Pichia pastoris* and* Aspergillus* sp. [191, 195]. In contrast to homologous laccase production, in which copper addition often affects laccase gene expression, the increased laccase production by copper addition is probably related to improving folding of the active laccase in heterologous production [195]. The importance of adequate copper concentration for proper laccase folding was further corroborated by studies in which two genes related to copper-trafficking in* Trametes versicolor* were overexpressed in* S. cerevisiae* expressing* T. versicolor lacIII* gene; the heterologous laccase production by* S. cerevisiae* was improved up to 20-fold [196]. The effect was suggested to result from more efficient transport of copper to the Golgi compartment [196].

Directed evolution has also been used for improving heterologous laccase production. Mutations in the* Myceliophthora thermophila* laccase gene resulted in the highest reported laccase production level in* S. cerevisiae* [197]. The most important obstacles to commercial application of laccases are the lack of sufficient enzyme stocks and the cost of redox mediators. Thus, efforts have to be made in order to achieve cheap overproduction of these biocatalysts in heterologous hosts and also their modification by chemical means of protein engineering to obtain more robust and active enzymes.

## 8. Mode of Action

Laccases are mostly extracellular glycoproteins [83] and are multinuclear enzymes [69] with molecular weights between 60 and 80 kDa [83]. Most monomeric laccase molecules contain four copper atoms in their structure that can be classified in three groups using UV/visible and electron paramagnetic resonance (EPR) spectroscopy [198]. The type I copper (T1) is responsible for the intense blue colour of the enzymes at 600 nm and is EPR-detectable, the type II copper (T2) is colourless but is EPR-detectable, and the type III copper (T3) consists of a pair of copper atoms that give a weak absorbance near the UV spectrum but no EPR signal [199]. The T2 and T3 copper sites are close together and form a trinuclear centre [198] that are involved in the catalytic mechanism of the enzyme [199].

Laccase only attacks the phenolic subunits of lignin, leading to C*α* oxidation, C*α*-C*β* cleavage, and aryl-alkyl cleavage. Laccases are able to reduce one molecule of dioxygen to two molecules of water while performing one-electron oxidation of a wide range of aromatic compounds [8], which includes polyphenols [200], methoxy-substituted monophenols, and aromatic amines [201]. This oxidation results in an oxygen-centred free radical, which can then be converted into a second enzyme-catalysed reaction to quinone. The quinone and the free radicals can then undergo polymerization [8]. Laccases are similar to other phenol-oxidising enzymes, which preferably polymerise lignin by coupling of the phenoxy radicals produced from oxidation of lignin phenolic groups [202]. Due to this specificity for phenolic subunits in lignin and their restricted access to lignin in the fibre wall, laccase has a limited effect on pulp bleaching [200]. The substrate range of laccase can be extended to nonphenolic subunits of lignin by the inclusion of a mediator such as 2, 2′-azinobis-(3-ethylbenzthiazoline-6-sulfonate) (ABTS).

## 9. Biotechnological Applications of Laccase

Laccases of fungi are of particular interest with regard to potential industrial applications because of their capability to oxidize a wide range of industrially relevant substrates. Oxidation reactions are comprehensively used in industrial processes, for instance, in the textile, food, wood processing and pharmaceutical and chemical industries. Enzymatic oxidation is a potential substitute to chemical methods since enzymes are very specific and efficient catalysts and are ecologically sustainable. Laccases are currently studied intensively for many applications and they are already used in large scale in the textile industry. Together with low molecular weight redox-mediator compounds, laccases can generate a desired worn appearance on denim by bleaching indigo dye [203]. The potential use of laccase for bleaching has been investigated and this has even led to the esoteric suggestion of using laccase in the presence of hydroxyl stilbenes as hair dyes [204]. Another potential environmental application for laccases is the bioremediation of contaminated soils as laccases are able to oxidize toxic organic pollutants, such as polycyclic aromatic hydrocarbons [205] and chlorophenols [150]. The most useful method for this application would probably be inoculating the soil with fungi that are efficient laccaseproducers because the use of isolated enzymes is not economically feasible for soil remediation in large scale. The current practical applications of the use of laccase have led to a search for source of the enzyme from white-rot fungi and the use of mediators, which promote or facilitate enzyme action.

## 10. Laccases Role in Bioremediation

One of the major environmental problems, faced by the world today, is the contamination of soil, water, and air by toxic chemicals. With industrialization and the extensive use of pesticides in agriculture, the pollution of the environment with mandate organic compounds has become a serious problem. Eighty billion pounds of hazardous organopollutants are produced annually in the United States and only 10% of these are disposed of safely [206]. Certain hazardous compounds, such as polycyclic aromatic hydrocarbons (PAH), pentachlorophenols (PCP), polychlorinated biphenyls (PCB), 1,1,1-trichloro-2,2-bis(4-chlorophenyl) ethane (DDT), benzene, toluene, ethylbenzene, and xylene (BTEX) as well as trinitrotoluene (TNT), are persistent in the environment and are known to have carcinogenic and/or mutanogenic effects. The ability of fungi to transform a wide variety of hazardous chemicals has aroused interest in using them in bioremediation [207]. Enzymatic treatment is currently considered an alternative method for the removal of toxic xenobiotics from the environment [150].

### 10.1. Degradation of Xenobiotics

Laccases exhibit broad substrate specificity and is thus able to oxidize a broad range of xenobiotic compounds including chlorinated phenolics [208], pesticides [209], and polycyclic aromatic hydrocarbons [210]. Moreover, polycyclic aromatic hydrocarbons, which arise from natural oil deposits and utilization of fossil fuels, were also found to be degraded by laccases [211]. Laccase purified from a strain of* Coriolopsis gallica* oxidized carbozole,* N-*ethylcarbozole, fluorine, and dibenzothiophene in presence of 1-hydroxybenzotriazole and 2.2′-azinobis (3-ethylbenzthiazoline)-6-sulfonic acid as free radical mediators [212]. Laboratory experiments have demonstrated that phenols and aromatic amines may be removed from water by the application of laccase [213]. The underlying mechanism of the removal involves enzymatic oxidation of the pollutants to free radicals or quinones that undergo polymerization and partial precipitation [213]. Laccase from white-rot fungus,* Trametes hirsuta*, has been used to oxidize alkenes [214]. The oxidation is the effect of a two-step process in which the enzyme first catalysed the oxidation of primary substrate, a mediator added to the reaction, and then the oxidized mediator oxidizes the secondary substrate, the alkene, to the corresponding ketone or aldehyde. In addition to substrate oxidation, laccase can also immobilize soil pollutants by coupling to soil humic substances—a process analogous to humic acid synthesis in soils [215]. The xenobiotics that can be immobilized in this way include phenolic compounds including chlorinated phenols and anilines such as 3, 4-dichloroaniline, 2, 4, 6-trinitrotoluene, or chlorinated phenols [216]. The immobilization lowers the biological availability of the xenobiotics and thus their toxicity. A laccase produced in the yeast,* Pichia pastoris*, was engineered by site-directed mutagenesis to improve the rate of electron transfer between the copper-containing active site of laccase and an electrode [217]. Thus laccase may be usefully engineered to improve the efficiency of particular bioremediation processes.

### 10.2. Decolourisation of Dyes

The textile industry accounts for two-thirds of the total dyestuff market and consumes large volumes of water and chemicals for wet processing of textiles [218]. The chemical reagents used are very diverse in chemical composition, ranging from inorganic compounds to polymers and organic products [219]. There are about 1,00,000 commercially available dyes with over 7 × 10^5^ tonnes of dyestuff produced annually [220]. Due to their chemical structure dyes are resistant to fading on exposure to light, water, and different chemicals and most of them are difficult to decolourise due to their synthetic origin.

Government legislation is becoming more and more stringent, especially in the more developed countries, regarding the removal of dyes from industrial effluents [221]. Concern arises as several dyes are made from known carcinogens such as benzidine and other aromatic compounds [222]. Most currently existing processes to treat dye wastewater are ineffective and not economical. Therefore, the development of processes based on laccases seems an attractive solution due to their potential in degrading dyes of diverse chemical structure [223] including synthetic dyes currently employed in the industry [224]. The use of laccase in the textile industry is growing very fast since, besides decolorizing textile effluents as commented above, laccase is used to bleach textiles and even to synthesize dyes [225].* Flavodon flavus* decolourized several synthetic dyes such as Azure B and Brilliant Blue R in low nitrogen medium [226]. Alternatively, laccase, along with stabilizers, may be suitable for treatment of wastewater [227, 228]. Partial decolorization of two azo dyes and complete decolorization of two triphenylmethane dyes (bromophenol blue and malachite green) was achieved by cultures of* Pycnoporus sanguineus* producing laccase as the sole phenoloxidase [120, 229]. Saratale et al. [230] demonstrated employing HPLC analysis, the degradation of the dye “Navy blue HER” by fungus* Trichosporon beigelii* NCIM-3326 after one day under static conditions for the identification of metabolic products from dye degradation. Enayatizamir et al. [231] observed degradation of 92% in the Azo Black Reactive 5 dye by* P. chrysosporium* after 3 days of treatment.* P. chrysosporium* URM 6181 and* Curvularia lunata* URM 6179 strains decolourize effluent containing textile indigo dye by approximately 95% for 10 days of treatment [232]. Laccase purified from the fungus* Trametes hirsuta* was able to degrade triarylmethane, indigoid, azo, and athraquinonic dyes used in dyeing textiles [233] as well as 23 industrial dyes [234].

### 10.3. Effluent Treatment

Laccases from fungi offer several advantages of great interest to biotechnological applications of industrial effluent treatment. As they exhibit broad substrate specificity, they can bleach Kraft pulp or detoxify agricultural byproducts including olive mill wastes or coffee pulp [235]. Laccase of an isolate of the fungus,* Flavodon flavus,* was shown to decolourize the effluent from a Kraft paper mill bleach plant [226]. Laccase purified from white-rot basidiomycete,* Trametes villosa, *degrades bisphenol A, an endocrine-disrupting chemical [225]. Nonylphenols have increasingly gained attention because of their potential to mimic the action of natural hormones in vertebrates [236]. They result from incomplete biodegradation of nonylphenol polyethoxylates (NPEOs), which have been widely used as nonionic surfactants in industrial processes. Both nonylphenols and NPEOs are discharged into the environment, mainly due to incomplete removal of wastewater treatment facilities [236]. Nonylphenols are more resistant to biodegradation than their parent compound and hence are found worldwide in wastewater treatment plant effluents and rivers [237]. Due to their hydrophobicity, they tend to be absorbed onto surface water particles and sediments and accumulate in aquatic organisms. Consequently, nonylphenols represent a serious environmental and human health risk. Laccases from aquatic hyphomycete,* Clavariopsis aquatica*, have proved to degrade xenoestrogen nonylphenol [238]. In addition to the potential role of such degradation processes for natural attenuation processes in freshwater environments, this enzyme laccase also offers new perspectives for biotechnological applications such as wastewater treatment.

### 10.4. Laccases: Pulp and Paper

Pulp bleaching is currently achieved by treating pulps with chlorine-based chemicals. This results in the formation of chlorinated aliphatic and aromatic compounds that could be acutely toxic, mutagenic, and carcinogenic [239]. In recent years there have been intensive studies performed to develop enzymatic, environmentally benign, bleaching technologies [239]. The use of laccase-mediated systems has shown potential for the biobleaching of pulp, but the feasibility of its use is hindered by the lack of an inexpensive mediator [239]. The bioremediatory role of laccases in the pulp and paper industry is hindered by the alkalinity of the effluent. Thus several researchers have spent considerable effort in identifying laccases that could be suitable for this type of remediation. The laccase from* Coriolopsis gallica* has been implicated in the decolourisation of alkaline effluents such as the effluent from the pulp and paper industry [240]. Laccases have also been shown to be applicable to the bioremediation of pulp and paper industry waste by effecting direct dechlorination [241] and the removal of chorophenols and chlorolignins from bleach effluents [242]. Other uses of laccases for the pulp and paper industry include reduction of the kappa number of pulp [243] and an improvement in the paper making properties of pulp [244]. Fungal laccases can be used for the treatment of effluents from pulp mills or from other industries containing chlorolignins or phenolic compounds [245]. Laccases render phenolic compounds less toxic via degradation or polymerization reactions and/or cross-coupling of pollutant phenol with naturally occurring phenols [170].

### 10.5. Laccases: Biosensors and Biofuel Cells

The use of laccase in biosensor technology is mainly attributed to its broad substrate range allowing for the detection of a broad range of phenolics; this does however disallow the detection of specific constituents [246, 247]. Biosensors that utilize laccase include an electrode that may be used for the detection of phenols, such as catechols in tea [247], phenolic compounds in wine, and lignins and phenols in wastewaters [248]. Fogel and Limson [246] developed a rapid, simple method of electrochemically predicting a given phenolic substrate's ability by amperometric laccase biosensors. Novel biosensors have been developed using beneficial properties of laccase, such as the potentiometric immunosensor for the detection of antigens [248]. Laccase has displayed a significant potential for its use in biofuel cells [249]. The major reason for this interest is the use of oxygen as a substrate, which is converted into water. The obvious advantage of this is the potential use in nanotechnology for medical applications in living animals since oxygen may be scavenged from the bloodstream, while the byproduct (water) is benign. Slomczynski et al. [250] developed a zinc-laccase biofuel cell adapting the zinc-air cell design configuration. Unlike most biofuel cells, this zinc-laccase cell operated under open ambient conditions. In this single chamber, membraneless cell design was utilized and laccase biocatalyst was left to be freely suspended (i.e., not immobilized) in quasineutral potassium dihydrogen phosphate buffer (pH 6.5) electrolyte. Despite its simple design features and not operating under controlled conditions, the zinc-laccase system studied demonstrated power output of comparable performance to biofuel cell system utilizing a much more complex design immobilized enzyme and electron transfer mediator, controlled temperature and humidity, oxygenated electrolyte, and so forth [250]. The drawback of using laccase in this technology is its inability to reduce oxygen at the physiological pH of blood, a technical hurdle that must be overcome [251].

### 10.6. Food and Beverage Industry

The beverage industry is also set to be a benefactor of laccase. Laccase may prevent undesirable changes such as discoloration, clouding, haze, or flavour changes in beer, fruit juices, and wine, improving their shelf life by removing phenols such as coumaric acids, flavans, and anthocyanins [150, 251]. The practical applications of laccases have led to a search for sources of the enzyme from white-rot fungi and the use of mediators, which promote or facilitate enzyme action. This review summarizes the available data about the biological properties of fungal laccases, their occurrence, and biotechnological applications. It is clear from the forgone survey of literature that focus has been paid to a few laccase producing fungi, particularly* Phanerochaete chrysosporium, Trametes versicolor, Pleurotus ostreatus*, and so forth, but there are still large numbers of fungal organisms not covered for laccase production. Search and screening of uncovered fungal organisms are needed along with the known and reference culture to select the potential culture with production of laccase in larger amounts. Further understanding of kinetic parameters of laccases will be useful to application of laccases for practical purposes.

## 11. Conclusion

Because of their specific nature, laccases are receiving much attention from researchers around the globe. The interest in utilizing laccases for biotechnological applications has increased rapidly since the discovery of these enzymes in white-rot fungi. Emerging technologies include selective delignification for production of cellulosics in pulp bleaching, conversion of lignocellulosics into feed and biofuel, and treatment of environmental pollutants and toxicants generated in various industrial processes. Therefore, laccases have been widely studied for various applications, including the functionalization of lignocellulosic materials, wood fiber modification, and the remediation of soil and contaminated effluents as well as their use in biosensors. This review shows that laccase has a great potential application in environment protection. However, much more research is required to make use of laccases to protect environment and other industrial applications.

## Figures and Tables

**Table 1:** Kinetic constants of laccases at specified pH.

Substrate	*K*_*M*_	*k*_cat_	pH	Laccase	Reference
	(M)	(min^−1^)			
Guaiacol	1200	150	6	*Pleurotus ostreatus* POXC	[165]
	3100	n.r*	6	*Pleurotus ostreatus* POXA2	[165]
	400	n.r	6	*Chaetomium thermophilum *	[174]
	5120	115	3.4	*Trametes trogii* POXL3	[148]
	510	n.r	4.5	*Gaeumannomyces graminis *	[249]
	36	10800	3	*Trametes pubescens* LAP2	[181]
	66	6800	6.5	*Pleurotus sajor-caju* Lac4	[185]

ABTS	58	2700	5.3	*Trametes villosa* Lcc1	[169]
	52	n.r	5.3	*Rhizoctonia solani* Lcc4	[169]
	90	350000	3	*Pleurotus ostreatus* POXA1	[165]
	120	n.r	3	*Pleurotus ostreatus* POXA2	[165]
	280	57000	3	*Pleurotus ostreatus* POXC	[165]
	190	n.r	6	*Chaetomium thermophilum *	[174]
	30	198	3.4	*Trametes trogii* POXL3	[148]
	32	n.r	3	*Panaeolus sphinctrinus *	[83]
	41	n.r	5	*Coprinus friesii *	[83]
	23	1090	5.5	*Coprinus cinereus* Lcc1	[50]
	14	41400	3	*Trametes pubescens* LAP2	[81]
	45	620	5.5	*Trichophyton rubrum *	[175]
	55	n.r	4	*Pycnoporus cinnabarinus *	[184]
	2500	74000	3.3	*Pleurotus sajor-caju* Lac4	[185]
	290	790	6	*Myceliophthora thermophila *	[196]

Syringalda-zine	3.9	3000	5.3	*Trametes villosa* Lcc1	[169]
	28	n.r	5.3	*Rhizoctonia solani* Lcc4	[169]
	20	23000	6	*Pleurotus ostreatus* POXC	[165]
	130	28000	6	*Pleurotus ostreatus* POXA1	[165]
	140	n.r	6	*Pleurotus ostreatus* POXA2	[165]
	34	n.r	6	*Chaetomium thermophilum *	[174]
	26	180	5.5	*Coprinus cinereus* Lcc1	[50]
	6	16800	4.5	*Trametes pubescens* LAP2	[181]
	280	35000	6.5	*Pleurotus sajor-caju* Lac4	[185]
	1.6	2100	6	*Myceliophthora thermophila *	[196]

2,6-DMP	100	n.r	3.5	*Botrytis cinerea *	[251]
	230	430	5	*Pleurotus ostreatus* POXC	[165]
	2100	21000	5	*Pleurotus ostreatus* POXA1	[165]
	740	n.r	6.5	*Pleurotus ostreatus* POXA2	[165]
	410	109	3.4	*Trametes trogii* POXL3	[148]
	96	n.r	6	*Chaetomium thermophilum *	[174]
	26	n.r	4.5	*Gaeumannomyces graminis *	[249]
	72	24000	3	*Trametes pubescens* LAP2	[181]
	120	58000	6	*Pleurotus sajor-caju* Lac4	[185].

*n.r: not reported.

ABTS: 2,2′-azinobis-(3-ethylbenzthiazoline-6-sulphonate).

2,6-DMP: 2,6-dimethoxyphenol.

**Table 2:** Laccase genes that have been shown to encode a biochemically characterized laccase protein [252].

Organism	Gene		Protein encoded by the gene		
	Name	E MBL	Length	MW^+^	pI
		Acc. Number	(aa)**	(kDa)	
*Ceriporiopsis subvermispora *	*lcs-1 *	AY219235	519	79	3.6
*Coprinus cinereus *	*lcc1 *	AF118267	539	63	3.7–4.0
*Cryptococcus neoformans *	*CNLAC1 *	L22866	624	75	n.d.*
*Gaeumannomyces graminis *	*LAC2 *	AJ417686	577	70	5.6
*Marasmius quercophilus *	*lac1 *	AF162785	517	62	3.6
*Myceliophthora thermophila *	*lcc1 *	AR023901	619	80	4.2
*Neurospora crassa *	2 alleles	M18333-4	619	64	6.8
*Phlebia radiata *X52134	*lac1 *	—	548	64	3.5
*Pleurotus ostreatus *	*poxa1b *	AJ005017	533	62	6.9
*Pleurotus ostreatus *	*poxc *	Z49075	533	67	4.7
Basidiomycete PM1(CECT2971)	*lac1 *	Z12156	517	64	3.6
*Podospora anserina *	*lac2 *	Y08827	621	70	7.10
*Populus euramericana *	*lac90 *	Y13772	574	90	9.2
*Rhizoctonia solani *	*lcc4 *	Z54277	530	66	7.5
*Streptomyces lavendulae *	*— *	AB092576	631	73	n.d.*
*Trametes pubescens *	*lap2 *	AF414807	523	65	2.6
*Trametes trogii *	*lcc1 *	Y18012	496	70	3.3–3.6
*Trametes versicolor *	*lccI *	L49376	519	67	n.d.*
*Trametes versicolor *	*lcc2 *	U44430	520	64	3.1–3.3
*Trametes villosa *	*lcc1 *	L49377	520	63	3.5
*Trametes villosa *	*lcc2 *	AY249052	519	63	6.2–6.8

*n.d.: not determined.

^+^Molecular weights determined by SDS-PAGE.

**Amino acids.

**Table 3:** Laccase production in heterologous hosts [252].

Laccase gene	Production host	Laccase production (mgL^−1^)*
*Ceriporiopsis subvermispora lcs-1 *	*Aspergillus nidulans *	1.5
	*Aspergillus niger *	1.5
*Coprinus cinereus lcc1 *	*Aspergillus oryzae *	135
*Myceliophthora thermophila lcc1 *	*Aspergillus oryzae *	19
	*Saccharomyces cerevisiae *	18
*Phlebia radiata lac1 *	*Trichoderma reesei *	20
*Pleurotus sajor-caju lac4 *	*Pichia pastoris *	4.9
*Pycnoporus cinnabarinus lac1 *	*Pichia pastoris *	8
	*Aspergillus niger *	70
	*Aspergillus oryzae *	80

*The reported production levels have been obtained in shake flask cultivations, except in the case of *Phlebia radiata* laccase which was produced in a laboratory fermentor.
